# Crystal structure of 2-methyl-1*H*-imidazol-3-ium hydrogen oxalate dihydrate

**DOI:** 10.1107/S2056989016011038

**Published:** 2016-07-12

**Authors:** Mouhamadou Birame Diop, Libasse Diop, Laurent Plasseraud, Hélène Cattey

**Affiliations:** a Laboratoire de Chimie Minérale et Analytique (LACHIMIA), Département de Chimie, Faculté des Sciences et Techniques, Université Cheikh Anta Diop, Dakar, Senegal; b ICMUB UMR 6302, Université de Bourgogne, Faculté des Sciences, 9 avenue Alain Savary, 21000 Dijon, France

**Keywords:** crystal structure, organic salt, monosubstituted imidazolium, hydrogen oxalate, hydrogen bonds

## Abstract

Single crystals of the title mol­ecular salt, C_4_H_7_N_2_^+^·HC_2_O_4_^−^·2H_2_O, were isolated from the reaction of 2-methyl-1*H*-imidazole and oxalic acid in a 1:1 molar ratio in water. In the crystal, the cations and anions are positioned alternately along an infinite [010] ribbon and linked together through bifurcated N—H⋯(O,O) hydrogen bonds. The water mol­ecules of crystallization link the chains into (10-1) bilayers, with the methyl groups of the cations organized in an isotactic manner.

## Abstract 2

In the title mol­ecular salt 2-methyl-1*H*-imidazol-3-ium hydrogen oxalate dihydrate, N—H⋯(O,*O*) and O—H⋯O hydrogen bonds link the components into a bilayer-like assembly.

## Chemical context   

Imidazolium-type building blocks are useful in the field of crystal engineering (MacDonald *et al.*, 2001 ▸). With many possibilities of substitution (involving various positions around the five-membered ring) and *via* the propagation of multidirectional hydrogen-bonding inter­actions, they easily lead to the self-assembly of poly-dimensional packing net­works. In 2010, Callear and co-workers described various topologies based on imidazolium/di­carb­oxy­lic acid combinations and showed the crystal-packing effects of substitution in the imidazole ring (Callear *et al.*, 2010 ▸). In this context, and for some time, our group has focused on the contribution of the 2-methyl­imidazolium cation as a co-crystal in organic (Diop, Diop & Maris, 2016 ▸) and organic–inorganic hybrid salts (Diop, Diop & Maris, 2015 ▸; Diop, Diop, Plasseraud & Maris, 2015 ▸, 2016 ▸). Continuing our ongoing studies in this field, we report herein the crystal structure of a new hydrated organic salt, namely 2-methyl-1*H*-imidazol-3-ium hydrogen oxalate dihydrate, (I), isolated by reacting 2-methyl-1*H*-imidazole and oxalic acid in a 1:1 molar ratio in water.
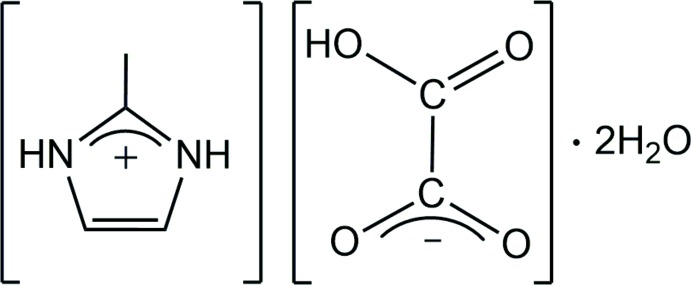


## Structural comments   

**Figure 1:**
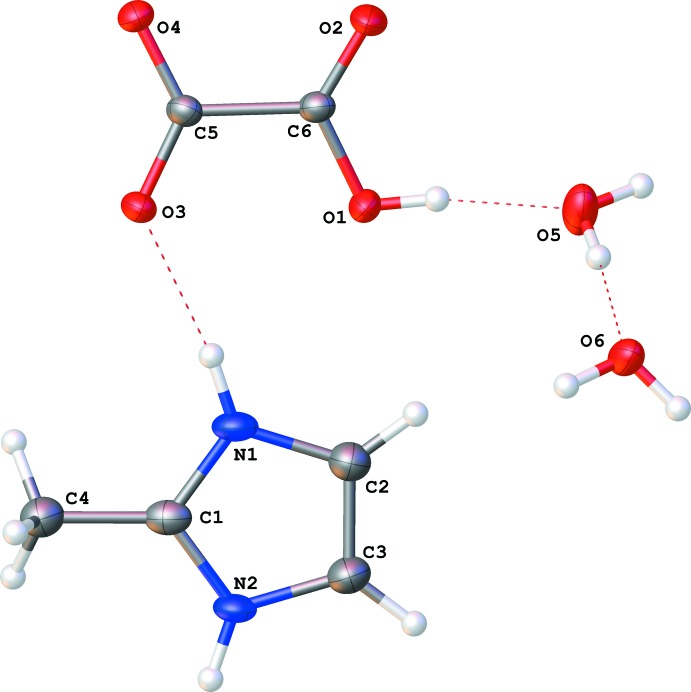
The mol­ecular structure of (I), showing the atom labelling. Displacement ellipsoids are draw at the 50% probability level.

The asymmetric unit of the title molecular salt (I) consists of four components, *i.e.* one 2-methyl-1*H*-imidazol-3-ium cation, one hydrogen oxalate anion and two solvent water mol­ecules (Fig. 1 ▸). The hydrogen oxalate anion is slightly twisted, with O1—C6—C5—O3 and O2—C6—C5—O4 torsion angles of 6.9 (3) and 7.3 (3)°, respectively. The C5—O3 and C5—O4 bonds are almost equal in length [1.249 (2) and 1.245 (2) Å, respectively], whereas C6—O2 is typical for a >C=O group [1.206 (2) Å] and C6—O1 has a normal C—OH bond length [1.306 (2) Å] (Adams, 1978 ▸).

## Supra­molecular features   

**Figure 2:**
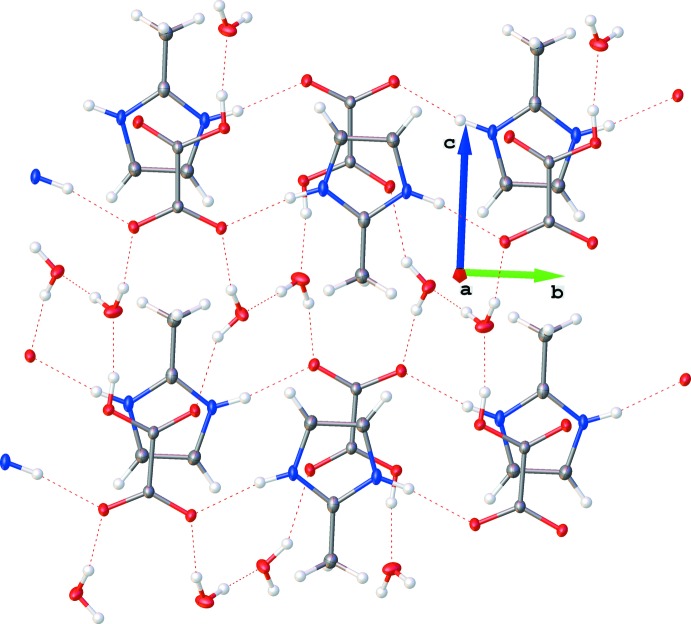
The crystal packing of the title salt, showing a two-dimensional bilayer-like arrangement through N—H⋯(O,O) and O—H⋯O inter­actions. H atoms not involved in hydrogen bonding have been omitted for clarity. Colour code: C dark grey, H light grey, O red and N blue.

Hydrogen-bonding inter­actions are listed in Table 1 ▸ and illustrated in Fig. 2 ▸. Both N—H groups of the imidazolium cation are involved in asymmetric bifurcated N—H⋯(O,O) hydrogen bonds with two distinct neighbouring hydrogen oxalate anions, which initiates the propagation of an infinite ribbon along the *b*-axis direction. Considering the orientation of the methyl groups of the cations along the ribbon, the sequence can be described as ‘isotactic’. The cations and anions are positioned alternately and are almost coplanar [dihedral angle between adjacent species = 1.15 (9)°].

As well as the cation-to-anion links, the OH group of the anion acts as a hydrogen-bond donor with one mol­ecule of water, which is also the donor for hydrogen-bond inter­actions with (i) a second mol­ecule of water and (ii) an O atom of a hydrogen oxalate anion involved in a neighbouring ribbon. The second water mol­ecule also bridges two distinct hydrogen oxalate anions through two O—H⋯O hydrogen bonds. Thus, all the O atoms of the hydrogen oxalate anions are involved in the hydrogen-bonding network.

The supra­molecular arrangement depicted in Fig. 2 ▸ relies on the contributions of the four components of (I) and can be described as resulting from three levels of organization: (i) C_4_H_7_N_2_^+^ and HC_2_O_4_^−^ assembled in infinite ribbons; (ii) parallel ribbons of C_4_H_7_N_2_^+^/HC_2_O_4_^−^ connected together by water mol­ecules, which leads to a staircase–sheet structure; (iii) sheets stacked in pairs which can be described as a two-dimensional bilayer-like arrangement propagating in (10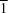). This final organization is again induced by the formation of hydrogen-bonding inter­actions between the water mol­ecules contained in each sheet. The inter-sheet distance is about 3.4 Å. Inter­estingly, all the methyl substituents of the imidazolium rings are oriented in the same direction along the *c* axis. Thus, the isotacticity observed at the ribbon level is also extended across the supra­molecular network.

## Database survey   

To date, 176 structures of hydrogen oxalates have been deposited in the Cambridge Structural Database (CSD; Groom *et al.*, 2016 ▸). Among these, five hits describe imidazolium salts or derivatives, *i.e.* imidazolium hydrogen oxalate [CSD refcodes MEQPAZ (MacDonald *et al.*, 2001 ▸) and MEQPAZ01 (Prasad *et al.*, 2002 ▸)], (*S*)-(+)-2-[2-(biphenyl-2-yl)-1-methyl­eth­yl]-4,5-di­hydro-1*H*-imidazolium hydrogen ox­alate (GAQTOI; Giannella *et al.*, 2005 ▸), 1,3-diisopropyl-4,5-di­methyl­imidazolium hydrogen oxalate (DOHTOK; Abu-Rayyan *et al.*, 2008 ▸), (*S*)-(−)-6-(4-bromo­phen­yl)-2,3,5,6-tetra­hydro­thia­zolo[2,3-*b*]imidazolium hydrogen oxalate (ROF­QAF; Minor & Chruszcz, 2008 ▸).

## Synthesis and crystallization   

Equimolar solutions of 2-methyl-1*H*-imidazole (6.51 g, 79.39 mmol) and H_2_C_2_O_4_·2H_2_O (10.00 g, 79.39 mmol) in water (100 ml) were mixed together at room temperature (301 K). Needle-shaped colourless crystals of (I) were obtained after one week by evaporation of the solvent at 333 K (yield 10.83 g, 65.5%).

## Refinement   

Crystal data, data collection and structure refinement details are summarized in Table 2 ▸. All H atoms on C, O and N atoms were placed at calculated positions using a riding model, with aromatic C—H = 0.95 Å and aromatic N—H = 0.88 Å, and with *U*_iso_(H) = 1.2*U*_eq_(C,N), or hy­droxy O—H = 0.84 Å, water O—H = 0.85 Å and methyl C—H = 0.98 Å, and with *U*_iso_(H) = 1.5*U*_eq_(O,C).

## Supplementary Material

Crystal structure: contains datablock(s) global, I. DOI: 10.1107/S2056989016011038/hb7595sup1.cif

Structure factors: contains datablock(s) I. DOI: 10.1107/S2056989016011038/hb7595Isup2.hkl

Supporting information file. DOI: 10.1107/S2056989016011038/hb7595Isup3.cml

CCDC reference: 1491387

Additional supporting information: crystallographic information; 3D view; checkCIF report

## Figures and Tables

**Table 1:** Hydrogen-bond geometry (Å, °)

*D*—H⋯*A*	*D*—H	H⋯*A*	*D*⋯*A*	*D*—H⋯*A*
N1—H1⋯O3	0.88	1.94	2.811 (2)	172
N1—H1⋯O1	0.88	2.50	2.991 (2)	116
N2—H2⋯O4^i^	0.88	1.97	2.842 (2)	169
N2—H2⋯O2^i^	0.88	2.49	2.977 (2)	116
O1—H1*A*⋯O5	0.84	1.69	2.5234 (19)	169
O6—H6*A*⋯O2^ii^	0.85	2.02	2.7893 (19)	150
O6—H6*B*⋯O3^iii^	0.85	1.87	2.700 (2)	166
O5—H5*A*⋯O6	0.85	1.82	2.672 (2)	176
O5—H5*B*⋯O4^iv^	0.85	1.88	2.720 (2)	167

Symmetry codes: (i) 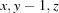; (ii) 
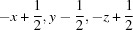
; (iii) 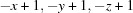; (iv) 
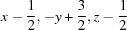
.

**Table 2:** Experimental details

Crystal data	
Chemical formula	C_4_H_7_N_2_^+^·C_2_HO_4_^−^·2H_2_O
*M*_r_	208.18
Crystal system, space group	Monoclinic, *P*2_1_/*n*
Temperature (K)	115
*a*, *b*, *c* (Å)	6.7139 (7), 9.5116 (7), 15.2115 (13)
β (°)	101.151 (6)
*V* (Å^3^)	953.07 (15)
*Z*	4
Radiation type	Mo *K*α
μ (mm^−1^)	0.13
Crystal size (mm)	0.30 × 0.10 × 0.08

Data collection	
Diffractometer	Bruker APEXII CCD
Absorption correction	Multi-scan (*SADABS*; Bruker, 2014 ▸)
*T*_min_, *T*_max_	0.691, 0.746
No. of measured, independent and observed [*I* > 2σ(*I*)] reflections	15445, 2187, 1258
*R*_int_	0.063
(sin θ/λ)_max_ (Å^−1^)	0.650

Refinement	
*R*[*F* ^2^ > 2σ(*F* ^2^)], *wR*(*F* ^2^), *S*	0.043, 0.115, 1.01
No. of reflections	2187
No. of parameters	135
H-atom treatment	H-atom parameters constrained
Δρ_max_, Δρ_min_ (e Å^−3^)	0.40, −0.23

Computer programs: *APEX2* and *SAINT* (Bruker, 2013 ▸), *SHELXS2013* (Sheldrick, 2008 ▸), *SHELXL2014* (Sheldrick, 2015 ▸) and *OLEX2* (Dolomanov *et al.*, 2009 ▸).

## supplementary crystallographic information

### Crystal data

**Table:**

C_4_H_7_N_2_^+^·C_2_HO_4_^−^·2H_2_O	*F*(000) = 440
*M**_r_* = 208.18	*D*_x_ = 1.451 Mg m^−^^3^
Monoclinic, *P*2_1_/*n*	Mo *K*α radiation, λ = 0.71073 Å
*a* = 6.7139 (7) Å	Cell parameters from 2578 reflections
*b* = 9.5116 (7) Å	θ = 3.5–26.4°
*c* = 15.2115 (13) Å	µ = 0.13 mm^−^^1^
β = 101.151 (6)°	*T* = 115 K
*V* = 953.07 (15) Å^3^	Needle, colourless
*Z* = 4	0.30 × 0.10 × 0.08 mm

### Data collection

**Table:**

Bruker APEXII CCD diffractometer	2187 independent reflections
Radiation source: X-ray tube, Siemens KFF Mo 2K-90C	1258 reflections with *I* > 2σ(*I*)
TRIUMPH curved crystal monochromator	*R*_int_ = 0.063
Detector resolution: 1024 x 1024 pixels mm^-1^	θ_max_ = 27.5°, θ_min_ = 2.5°
φ and ω scans'	*h* = −8→8
Absorption correction: multi-scan SADABS (Bruker, 2014) was used for absorption correction. wR2(int) was 0.0622 before and 0.0548 after correction. The Ratio of minimum to maximum transmission is 0.9269. The λ/2 correction factor is 0.00150.	*k* = −12→12
*T*_min_ = 0.691, *T*_max_ = 0.746	*l* = −19→19
15445 measured reflections	

### Refinement

**Table:**

Refinement on *F*^2^	0 restraints
Least-squares matrix: full	Hydrogen site location: mixed
*R*[*F*^2^ > 2σ(*F*^2^)] = 0.043	H-atom parameters constrained
*wR*(*F*^2^) = 0.115	*w* = 1/[σ^2^(*F*_o_^2^) + (0.0448*P*)^2^ + 0.3824*P*] where *P* = (*F*_o_^2^ + 2*F*_c_^2^)/3
*S* = 1.01	(Δ/σ)_max_ < 0.001
2187 reflections	Δρ_max_ = 0.40 e Å^−^^3^
135 parameters	Δρ_min_ = −0.23 e Å^−^^3^

### Special details

**Table:**

Geometry. All esds (except the esd in the dihedral angle between two l.s. planes) are estimated using the full covariance matrix. The cell esds are taken into account individually in the estimation of esds in distances, angles and torsion angles; correlations between esds in cell parameters are only used when they are defined by crystal symmetry. An approximate (isotropic) treatment of cell esds is used for estimating esds involving l.s. planes.

### Fractional atomic coordinates and isotropic or equivalent isotropic displacement parameters (Å^2^)

**Table:**

	*x*	*y*	*z*	*U*_iso_*/*U*_eq_	
O4	0.3344 (2)	0.84241 (13)	0.62704 (8)	0.0224 (3)	
O3	0.3377 (2)	0.60714 (13)	0.62300 (9)	0.0247 (3)	
O1	0.1928 (2)	0.60978 (13)	0.44891 (9)	0.0254 (4)	
H1A	0.1572	0.6196	0.3932	0.038*	
O6	0.3979 (2)	0.45757 (14)	0.22494 (10)	0.0314 (4)	
H6A	0.3724	0.3954	0.1842	0.047*	
H6B	0.4883	0.4270	0.2677	0.047*	
O2	0.2253 (2)	0.84212 (14)	0.44546 (9)	0.0311 (4)	
O5	0.1121 (3)	0.61142 (17)	0.27986 (9)	0.0357 (4)	
H5A	0.1986	0.5615	0.2599	0.054*	
H5B	0.0183	0.6360	0.2367	0.054*	
N2	0.2719 (2)	0.11229 (16)	0.54620 (11)	0.0231 (4)	
H2	0.2824	0.0244	0.5647	0.028*	
N1	0.2803 (2)	0.33729 (17)	0.54697 (11)	0.0245 (4)	
H1	0.2970	0.4248	0.5657	0.029*	
C5	0.3089 (3)	0.7264 (2)	0.58854 (12)	0.0186 (4)	
C6	0.2361 (3)	0.7327 (2)	0.48607 (12)	0.0187 (4)	
C1	0.3139 (3)	0.2239 (2)	0.59833 (14)	0.0244 (5)	
C2	0.2138 (3)	0.2951 (2)	0.45809 (14)	0.0287 (5)	
H2A	0.1789	0.3549	0.4074	0.034*	
C4	0.3880 (3)	0.2233 (2)	0.69571 (13)	0.0291 (5)	
H4A	0.3300	0.1428	0.7222	0.044*	
H4B	0.3472	0.3106	0.7215	0.044*	
H4C	0.5364	0.2160	0.7085	0.044*	
C3	0.2088 (3)	0.1547 (2)	0.45810 (14)	0.0296 (5)	
H3	0.1696	0.0954	0.4075	0.036*	

### Atomic displacement parameters (Å^2^)

**Table:**

	*U*^11^	*U*^22^	*U*^33^	*U*^12^	*U*^13^	*U*^23^
O4	0.0311 (8)	0.0146 (7)	0.0198 (7)	−0.0010 (6)	0.0005 (6)	−0.0023 (6)
O3	0.0375 (9)	0.0141 (7)	0.0196 (7)	0.0018 (6)	−0.0018 (6)	0.0018 (6)
O1	0.0417 (10)	0.0159 (7)	0.0160 (7)	−0.0014 (6)	−0.0010 (7)	−0.0013 (5)
O6	0.0439 (11)	0.0223 (8)	0.0224 (8)	0.0083 (7)	−0.0075 (7)	−0.0045 (6)
O2	0.0568 (11)	0.0135 (7)	0.0203 (8)	−0.0004 (7)	0.0009 (7)	0.0024 (6)
O5	0.0412 (11)	0.0421 (10)	0.0201 (8)	0.0125 (8)	−0.0030 (7)	−0.0046 (7)
N2	0.0282 (10)	0.0116 (8)	0.0285 (10)	−0.0004 (7)	0.0026 (8)	0.0012 (7)
N1	0.0280 (10)	0.0128 (8)	0.0322 (10)	0.0004 (7)	0.0043 (8)	−0.0006 (7)
C5	0.0192 (11)	0.0153 (9)	0.0200 (10)	0.0020 (8)	0.0010 (8)	0.0006 (8)
C6	0.0198 (10)	0.0145 (9)	0.0209 (10)	0.0008 (8)	0.0016 (8)	−0.0011 (8)
C1	0.0218 (11)	0.0178 (9)	0.0332 (12)	0.0000 (9)	0.0047 (9)	−0.0005 (9)
C2	0.0363 (13)	0.0221 (11)	0.0265 (11)	0.0009 (10)	0.0029 (10)	0.0007 (9)
C4	0.0348 (13)	0.0238 (10)	0.0282 (11)	−0.0002 (10)	0.0053 (10)	−0.0016 (9)
C3	0.0395 (14)	0.0205 (11)	0.0276 (12)	−0.0001 (10)	0.0033 (10)	−0.0028 (9)

### Geometric parameters (Å, º)

**Table:**

O4—C5	1.245 (2)	N1—H1	0.8800
O3—C5	1.249 (2)	N1—C1	1.325 (3)
O1—H1A	0.8400	N1—C2	1.398 (2)
O1—C6	1.306 (2)	C5—C6	1.542 (3)
O6—H6A	0.8499	C1—C4	1.469 (3)
O6—H6B	0.8501	C2—H2A	0.9500
O2—C6	1.206 (2)	C2—C3	1.336 (3)
O5—H5A	0.8500	C4—H4A	0.9800
O5—H5B	0.8500	C4—H4B	0.9800
N2—H2	0.8800	C4—H4C	0.9800
N2—C1	1.323 (2)	C3—H3	0.9500
N2—C3	1.385 (2)		
			
C6—O1—H1A	109.5	N2—C1—N1	107.91 (17)
H6A—O6—H6B	109.5	N2—C1—C4	126.36 (18)
H5A—O5—H5B	109.5	N1—C1—C4	125.72 (18)
C1—N2—H2	125.2	N1—C2—H2A	126.6
C1—N2—C3	109.64 (16)	C3—C2—N1	106.85 (18)
C3—N2—H2	125.2	C3—C2—H2A	126.6
C1—N1—H1	125.6	C1—C4—H4A	109.5
C1—N1—C2	108.83 (16)	C1—C4—H4B	109.5
C2—N1—H1	125.6	C1—C4—H4C	109.5
O4—C5—O3	127.68 (17)	H4A—C4—H4B	109.5
O4—C5—C6	115.41 (16)	H4A—C4—H4C	109.5
O3—C5—C6	116.90 (17)	H4B—C4—H4C	109.5
O1—C6—C5	113.79 (16)	N2—C3—H3	126.6
O2—C6—O1	124.38 (17)	C2—C3—N2	106.76 (18)
O2—C6—C5	121.81 (18)	C2—C3—H3	126.6
			
O4—C5—C6—O1	−173.95 (17)	C1—N1—C2—C3	0.0 (2)
O4—C5—C6—O2	7.3 (3)	C2—N1—C1—N2	0.0 (2)
O3—C5—C6—O1	6.9 (3)	C2—N1—C1—C4	179.0 (2)
O3—C5—C6—O2	−171.78 (19)	C3—N2—C1—N1	0.0 (2)
N1—C2—C3—N2	0.0 (2)	C3—N2—C1—C4	−179.1 (2)
C1—N2—C3—C2	0.0 (2)		

### Hydrogen-bond geometry (Å, º)

**Table:**

*D*—H···*A*	*D*—H	H···*A*	*D*···*A*	*D*—H···*A*
N1—H1···O3	0.88	1.94	2.811 (2)	172
N1—H1···O1	0.88	2.50	2.991 (2)	116
N2—H2···O4^i^	0.88	1.97	2.842 (2)	169
N2—H2···O2^i^	0.88	2.49	2.977 (2)	116
O1—H1*A*···O5	0.84	1.69	2.5234 (19)	169
O6—H6*A*···O2^ii^	0.85	2.02	2.7893 (19)	150
O6—H6*B*···O3^iii^	0.85	1.87	2.700 (2)	166
O5—H5*A*···O6	0.85	1.82	2.672 (2)	176
O5—H5*B*···O4^iv^	0.85	1.88	2.720 (2)	167

Symmetry codes: (i) *x*, *y*−1, *z*; (ii) −*x*+1/2, *y*−1/2, −*z*+1/2; (iii) −*x*+1, −*y*+1, −*z*+1; (iv) *x*−1/2, −*y*+3/2, *z*−1/2.
